# Supplementary figures and images for: Economic costs of invasive rodents worldwide: the tip of the iceberg
Source: PeerJ. 2023 Mar 24;11:e14935. doi: 10.7717/peerj.14935 (PMC10042159; doi:10.7717/peerj.14935)

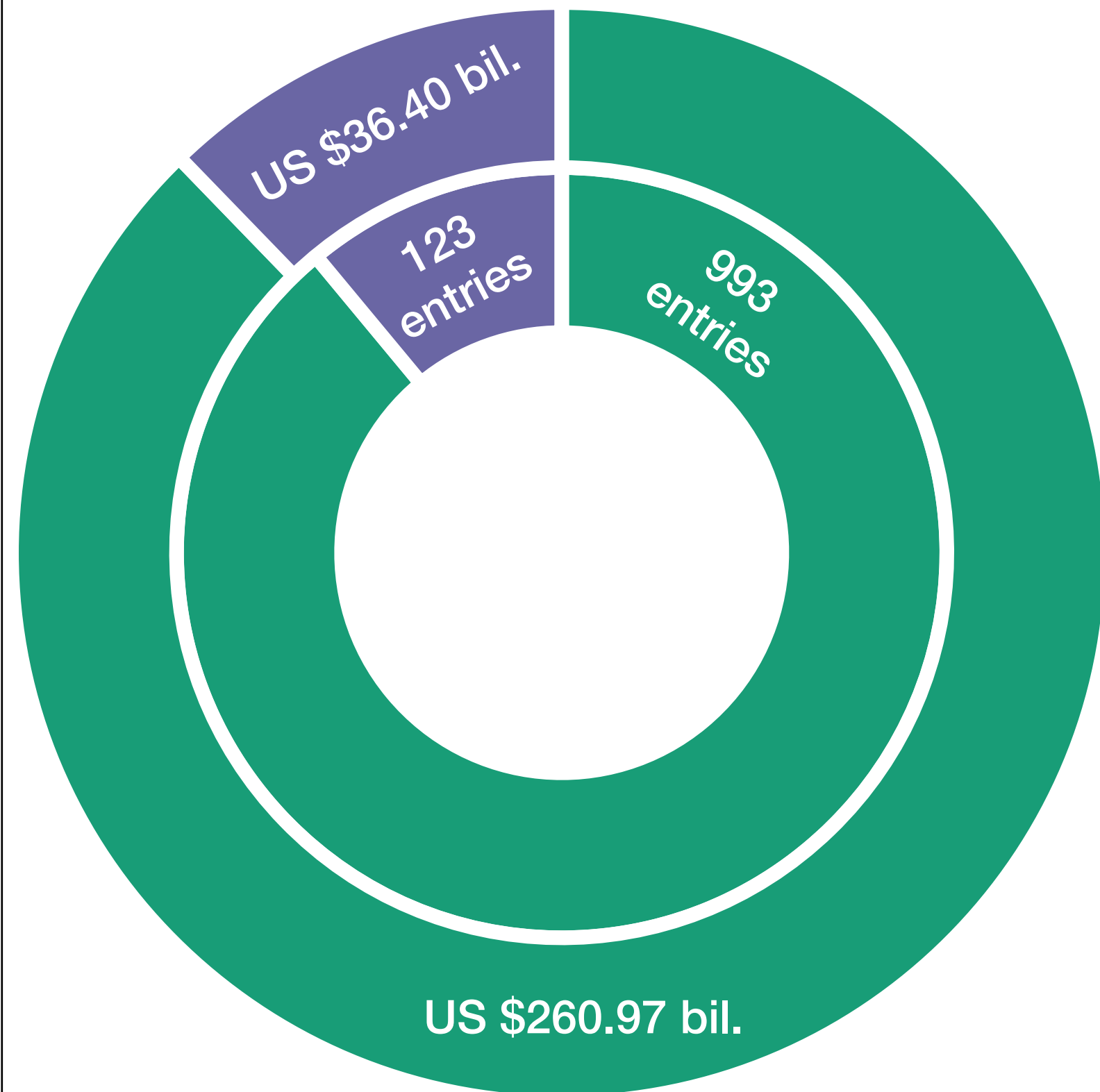

■ High

■ Low

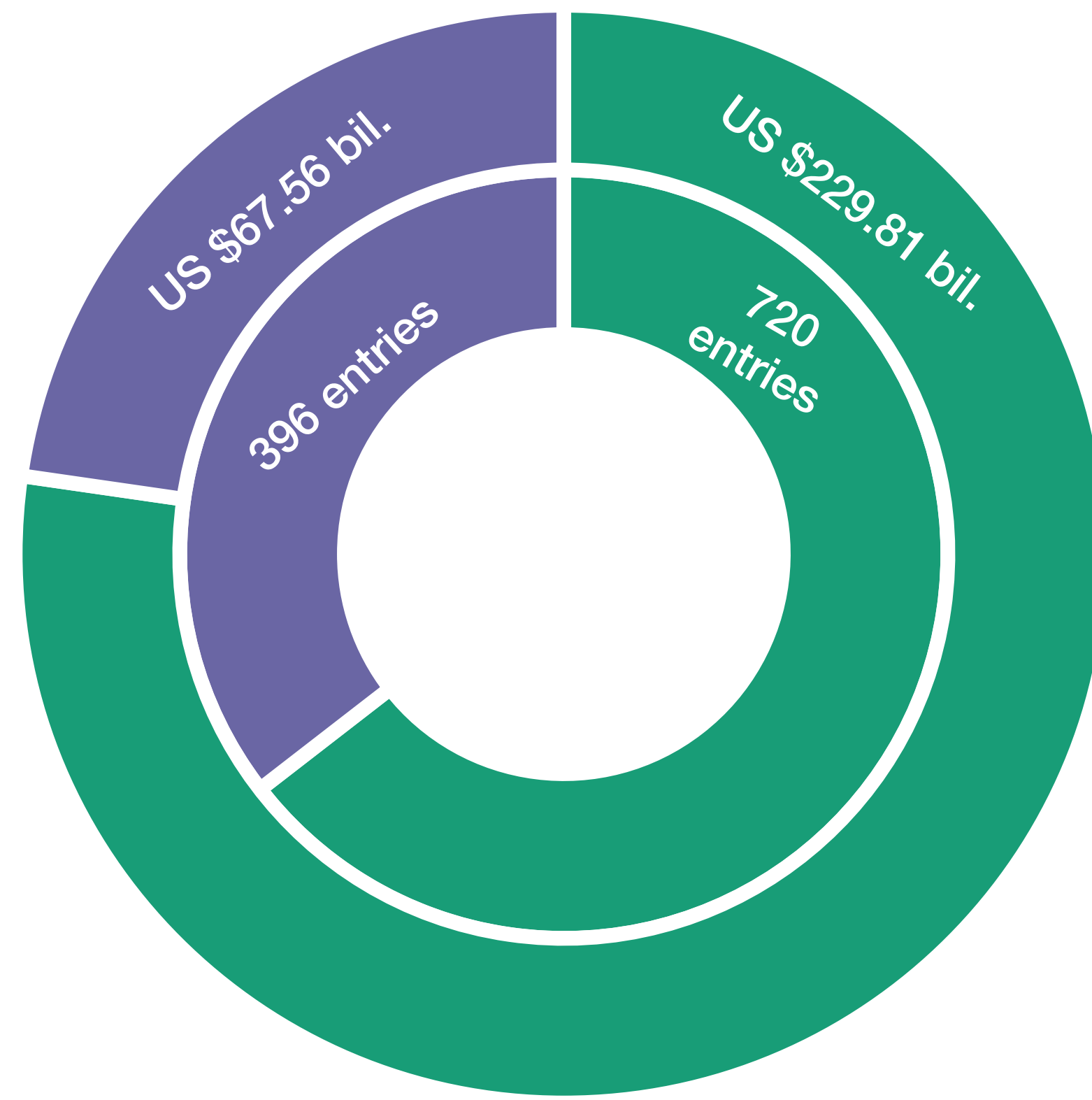

■ Observed

■ Potential

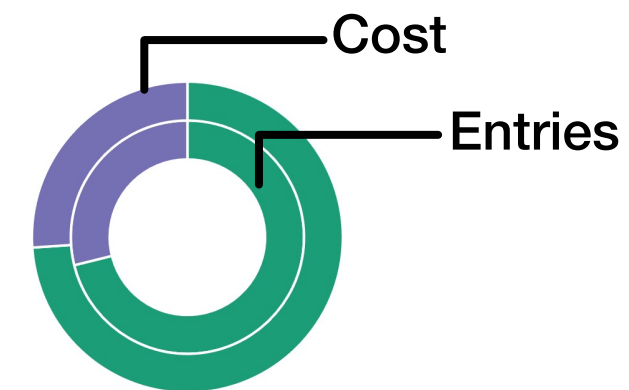

Supplement: Supplemental Information 2 — All details on the descriptive fields considered are available in Appendix 1. [file peerj-11-14935-s002.pdf]

(a)

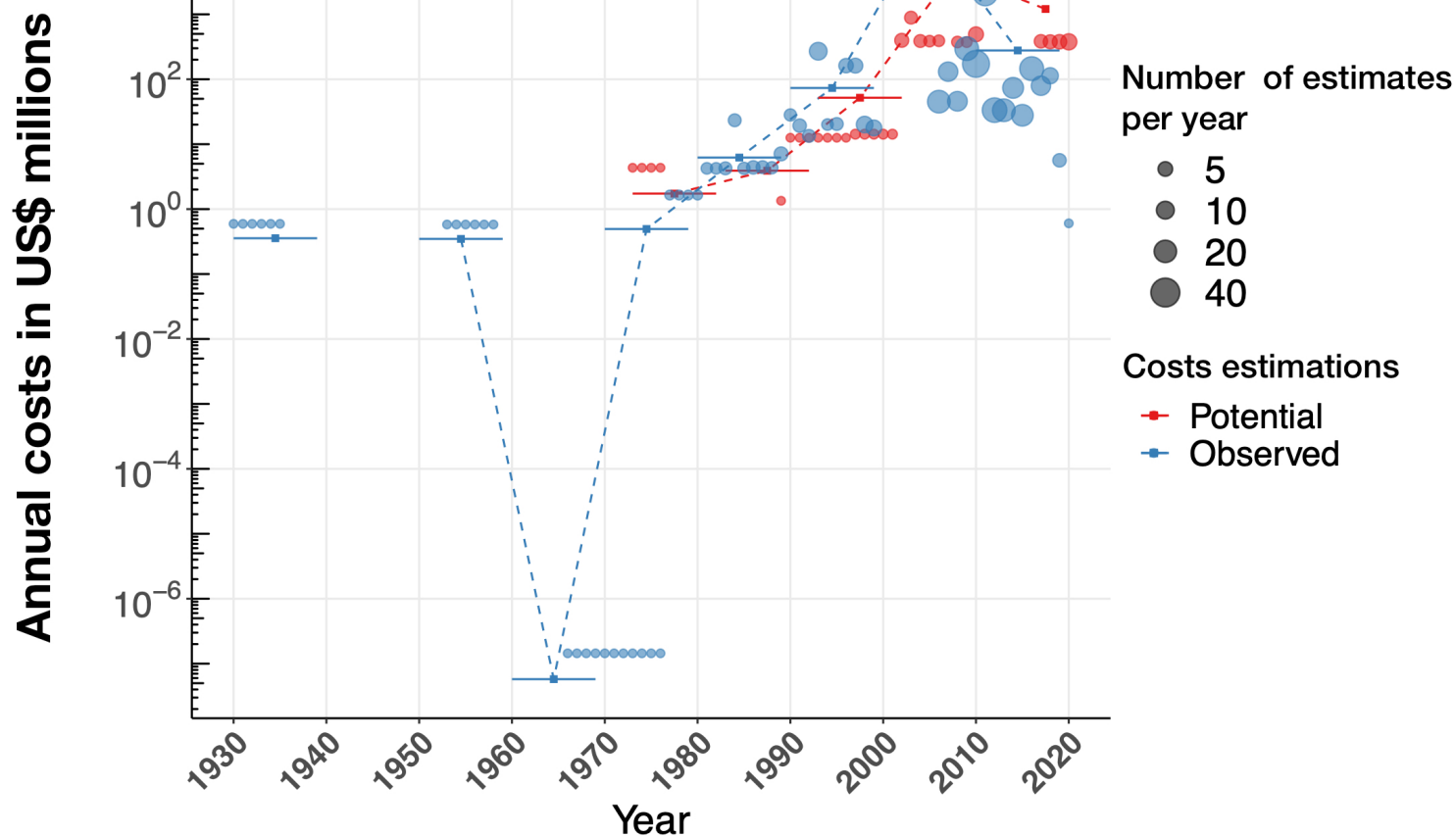

(b)

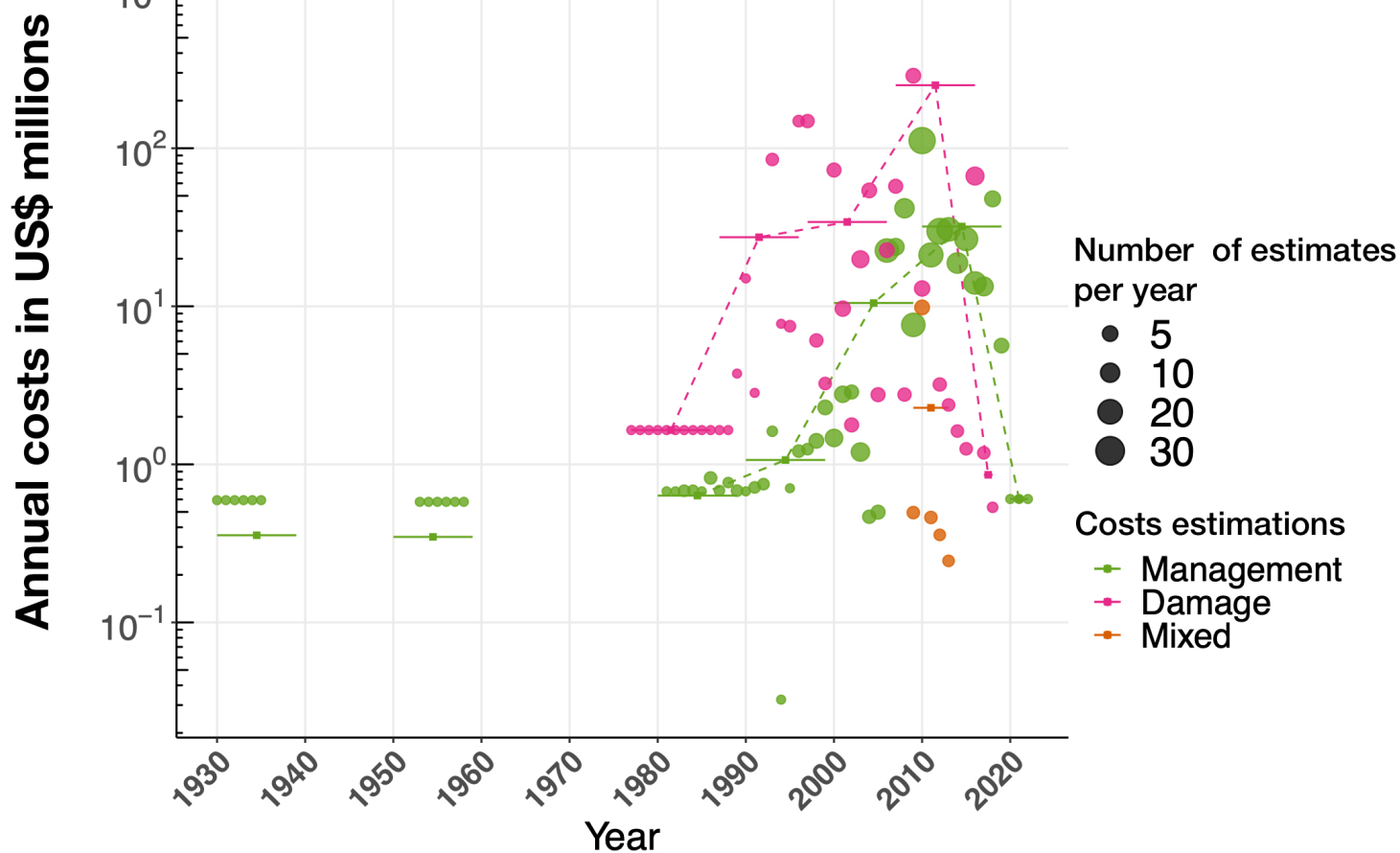

Supplement: Supplemental Information 3 — We considered (a) the expanded subset and (b) the conservative subset (see Fig. 1 and Appendix 1 for further details on the subset and filtering steps). In (a), the trend is described separately for potential and observed cost entries (see “Implementation” column; Appendix 1). In (b), trends are described separately for damage, management and mixed costs (see “Type of cost merged” column; Appendix 1). Costs are provided in millions of 2017 US$. The horizontal dotted lines represent annual averages over the decadal time period, solid bars represent 10-year means and filled circles represent annual costs scaled by size to match the number of entries. [file peerj-11-14935-s003.pdf]
