# Supplementary material for: Economic costs of invasive rodents worldwide: the tip of the iceberg
Source: PeerJ. 2023 Mar 24;11:e14935. doi: 10.7717/peerj.14935 (PMC10042159; doi:10.7717/peerj.14935)
Supplement: Supplemental Information 4 — For each line, the ‘Total_cost_estimate’ is calculated as the sum of cost values provided in the ‘Cost_estimate_per_year_2017_USD_exchange_rate’ column of the conservative subset. The number of annualized cost entries is provided in parenthesis. All details on the descriptive fields considered are provided in Appendix 1. [file peerj-11-14935-s004.pdf]

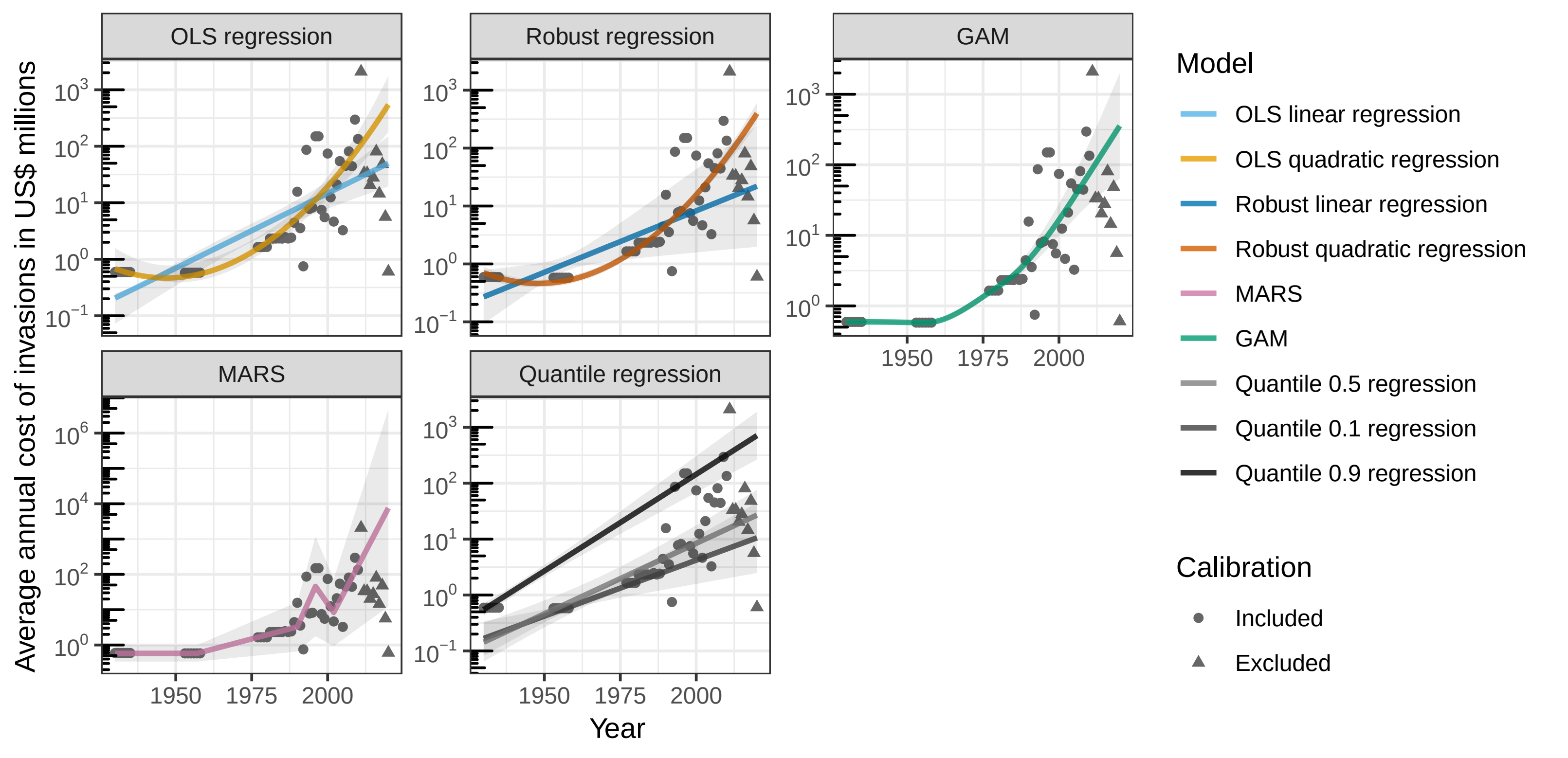
OLS regression
Robust regression
GAM
MARS
Quantile regression
Average annual cost of invasions in US$ millions
Year
1950
1975
2000
10^{-1}
10^{0}
10^{1}
10^{2}
10^{3}
10^{4}
10^{6}
Model
OLS linear regression
OLS quadratic regression
Robust linear regression
Robust quadratic regression
MARS
GAM
Quantile 0.5 regression
Quantile 0.1 regression
Quantile 0.9 regression
Calibration
Included
Excluded
