# Supplementary material for: Economic costs of invasive rodents worldwide: the tip of the iceberg
Source: PeerJ. 2023 Mar 24;11:e14935. doi: 10.7717/peerj.14935 (PMC10042159; doi:10.7717/peerj.14935)
Supplement: Supplemental Information 6 — Ordinary least squares (OLS), robust regression (RR), multivariate adaptive regression splines (MARS) and generalised additive model (GAM), as well as quantile regressions. Models considered annual total invasion costs as a function of time, between 1930 and 2020. [file peerj-11-14935-s006.docx]

**Appendix 6.** Estimates (2017 US$) and root mean square errors (RMSE) for models displayed in Appendix 5: ordinary least squares (OLS), robust regression (RR), multivariate adaptive regression splines (MARS) and generalised additive model (GAM), as well as quantile regressions. Models considered annual total invasion costs as a function of time, between 1930 and 2020.

| **Model** | **2020 cost (US$ million)** | **RMSE** |
| --- | --- | --- |
| **OLS (linear)** | 49.88 | 0.60 |
| **OLS (quadratic)** | 544.32 | 0.70 |
| **RR (linear)** | 21.97 | 0.63 |
| **RR (quadratic)** | 396.52 | 0.67 |
| **MARS** | 7,577.50 | 0.95 |
| **GAM** | 356.39 | 0.66 |
| **Quantile 0.1** | 10.54 | 0.75 |
| **Quantile 0.5** | 26.94 | 0.63 |
| **Quantile 0.9** | 710.61 | 1.12 |
